# Supplementary material for: Tear Fluid Biomarkers in Diabetic Ocular Surface Disease: A Systematic Review
Source: J Clin Med. 2025 Oct 1;14(19):6958. doi: 10.3390/jcm14196958 (PMC12524527; doi:10.3390/jcm14196958)
Supplement: Supplementary file 1 [file jcm-14-06958-s001.zip › jcm-3908906-supplementary.pdf]

Table S1. JBI Critical Appraisal Checklist for analytical cross sectional studies.

Autor, rok (Record)	Were the criteria for inclusion in the sample clearly defined?	Were the study subjects and the setting described in detail?	Was the exposure measured in a valid and reliable way?	Were objective, standard criteria used for measurement of the condition?	Were confounding factors identified?	Were strategies to deal with confounding factors stated?	Were the outcomes measured in a valid and reliable way?	Was appropriate statistical analysis used?	Overall appraisal:	Comments
Markoulli et al., 2017	Yes	Yes	Yes	Yes	Yes	No	Yes	Yes	Include	Small sample size, lack of full age control
Lagali et al., 2018	Yes	Yes	Yes	Yes	Yes	No	Yes	Yes	Include	No control of all confounding factors, but good methods and statistics
Tummanapalli et al., 2019	Yes	Yes	Yes	Yes	Yes	Yes	Yes	Yes	Include	Good study, large sample size, control of variables (age, HbA1c, BMI), appropriate statistical analysis
Byambajav et al., 2023	Yes	Yes	Yes	Yes	Yes	Yes	Yes	Yes	Include	Large sample size, several comparison groups, robust methodology, appropriate statistics
Zhou et al., 2024	Yes	Yes	Yes	Yes	Yes	Yes	Yes	Yes	Include	Very good prospective study, 70 participants, confounding factors controlled, robust statistical analysis
Liu et al., 2019	Yes	Yes	Yes	Yes	Yes	Yes	Yes	Yes	Include	Very good methodology, four comparison groups, DEWS II criteria applied, robust statistical analysis

Stuard et al., 2017	Yes	Yes	Yes	Yes	Yes	Yes	Yes	Yes	Include	Strong correlation between IGFBP-3 and corneal nerve loss, good methodology and statistical analysis
Britten-Jones et al., 2024	Yes	Yes	Yes	Yes	Yes	Yes	Yes	Yes	Include	Good study, NPY marker as a potential biomarker for microangiopathic complications in T1D
Cancarini et al., 2017	Yes	Yes	Yes	Yes	Yes	Yes	Yes	Yes	Include	Cross-sectional study, analysis of trace elements in tears and serum, appropriate methodology and statistics
Štorm et al., 2025	Yes	Yes	Yes	Yes	Yes	Yes	Yes	Yes	Include	Cross-sectional pilot study, modern markers of eye surface in T1D, small sample size
Qu et al., 2025	Yes	Yes	Yes	Yes	Yes	Yes	Yes	Yes	Include	Cross-sectional, MMP-9 in tears, good comparison of eye surface parameters, appropriate analysis
Qin et al., 2025 (29)	Yes	Yes	Yes	Yes	Yes	Yes	Yes	Yes	Include	Cross-sectional proteomic analysis of tear proteomes in adults with diabetes, robust methodology

Table S2. JBI checklist for quasi-experimental studies.

Study (Author, Year, Record)	Is it clear in the study what is the "cause" and what is the "effect"?	Was there a control group?	Were participants included in any comparisons similar?	Were the participants included in any comparisons receiving similar treatment/care, other than the exposure or intervention of interest?	Were there multiple measurements of the outcome, both pre and post the intervention/exposure?	Were the outcomes of participants included in any comparisons measured in the same way?	Were outcomes measured in a reliable way?	Was follow-up complete and if not, were differences between groups in terms of their follow-up adequately described and analyzed?	Was appropriate statistical analysis used?	Overall appraisal:
Nguyen-Khuong et al., 2015 (13)	Yes	Yes	Yes	Yes	No	Yes	Yes	Yes	Yes	Include
Tan et al., 2024 (16)	Yes	Yes	Yes	Yes	No	Yes	Yes	Yes	Yes	Include
